# Through the Burden: A Model of Self-Transcendence in in Family Caregivers of NH Residents

**DOI:** 10.1093/geroni/igaf122.4240

**Published:** 2025-12-31

**Authors:** Dalit Zaguri-Greener, Ruth Lopez, Anna Zisberg

**Affiliations:** University of Haifa, Haifa, Hefa, Israel; MGH Institute of Health Professions, Sharon, Massachusetts, United States; University of Haifa, Haifa, Hefa, Israel

## Abstract

**Background:**

Family caregivers of nursing home (NH) residents with advanced dementia often experience high emotional and logistical burden. While most models emphasize stress and coping, fewer address meaning-making and personal growth. This study applies Reed’s Self-Transcendence (ST) Theory to explore how caregiving may foster adaptation and meaning after placement.

**Objective:**

To examine whether perceptions of family-centered care predict caregiver burden and ST.

**Methods:**

Using a cross-sectional survey of 232 family caregivers of NH residents with advanced dementia, we examined the relationships between “Perception of Family-Centered Care” (basic care, symptom management, communication, trust, shared decision-making, and recognition of personhood), in NH staff, burden, and ST.

**Results:**

Structural equation modeling revealed that Perception of Family-Centered Care significantly predicted lower caregiver burden (β = –.611, p < .001). In turn, lower burden was associated with higher ST (β = –.52, p = .002). The direct path from Perception of Family-Centered Care to ST was nonsignificant (β = –.154, p = .218). However, the indirect effect from Perception of Family-Centered Care to ST through caregiver burden was significant (β = .67, 95% CI [.214 – 2.253], p = .000), indicating full mediation.

**Conclusions:**

Findings highlight the transformative nature of family caregiving in the NH setting. Recognizing family caregiving as a dynamic, transformative process can guide interventions that help family caregivers move through the burden-transcend toward meaning and connection. NH policies should consider integrating structured communication, family engagement, and decision-making support to reduce burden while fostering resilience, meaning-making, and role adaptation.

